# A Genome-Wide Analysis of Pathogenesis-Related Protein-1 (*PR-1*) Genes from *Piper nigrum* Reveals Its Critical Role during *Phytophthora capsici* Infection

**DOI:** 10.3390/genes12071007

**Published:** 2021-06-30

**Authors:** Divya Kattupalli, Asha Srinivasan, Eppurath Vasudevan Soniya

**Affiliations:** Transdisciplinary Biology, Rajiv Gandhi Centre for Biotechnology, Thiruvananthapuram 695014, Kerala, India; divyak@rgcb.res.in (D.K.); ashas@rgcb.res.in (A.S.)

**Keywords:** footrot, black pepper, promoter, cap domain, plant immunity, cis-regulatory element, biotic stress

## Abstract

Black pepper (*Piper nigrum* L.) is a prominent spice that is an indispensable ingredient in cuisine and traditional medicine. *Phytophthora capsici*, the causative agent of footrot disease, causes a drastic constraint in *P. nigrum* cultivation and productivity. To counterattack various biotic and abiotic stresses, plants employ a broad array of mechanisms that includes the accumulation of pathogenesis-related (*PR*) proteins. Through a genome-wide survey, eleven *PR-1* genes that belong to a CAP superfamily protein with a caveolin-binding motif (CBM) and a CAP-derived peptide (CAPE) were identified from *P. nigrum*. Despite the critical functional domains, *PnPR-1* homologs differ in their signal peptide motifs and core amino acid composition in the functional protein domains. The conserved motifs of *PnPR-1* proteins were identified using MEME. Most of the *PnPR-1* proteins were basic in nature. Secondary and 3D structure analyses of the *PnPR-1* proteins were also predicted, which may be linked to a functional role in *P. nigrum*. The GO and KEGG functional annotations predicted their function in the defense responses of plant-pathogen interactions. Furthermore, a transcriptome-assisted FPKM analysis revealed *PnPR-1* genes mapped to the *P. nigrum-P. capsici* interaction pathway. An altered expression pattern was detected for *PnPR-1* transcripts among which a significant upregulation was noted for basic *PnPR*-1 genes such as CL10113.C1 and Unigene17664. The drastic variation in the transcript levels of CL10113.C1 was further validated through qRT-PCR and it showed a significant upregulation in infected leaf samples compared with the control. A subsequent analysis revealed the structural details, phylogenetic relationships, conserved sequence motifs and critical cis-regulatory elements of *PnPR-1* genes. This is the first genome-wide study that identified the role of *PR-1* genes during *P. nigrum-P. capsici* interactions. The detailed in silico experimental analysis revealed the vital role of *PnPR-1* genes in regulating the first layer of defense towards a *P. capsici* infection in Panniyur-1 plants.

## 1. Introduction

Plant immunity involves multiple layers of defense responses. The first layer of defense is triggered by the detection of microbe-associated molecular patterns (MAMPs) through plant-, pathogen- or pattern-recognition receptors (PRRs), which activate PAMP-/pathogen-/pattern-triggered immunity (PTI) [1]. The defense machinery of plants has been forced to evolve continuously to combat a wide range of abiotic and biotic stress factors. These challenges activate an array of induced mechanisms such as a hypersensitive response (HR), which involves a series of events including the production of reactive oxygen species (ROS) and the synthesis of antimicrobial molecules and pathogenesis-related (PR) proteins. PR proteins induce programmed cell death, which inhibits the spread of infection contributing to a systemic acquired resistance (SAR) [2,3]. In the second layer of defense, pathogens suppress PTI by secreting the effector proteins that are later recognized by plant resistance (R) proteins leading to an effector-triggered immunity [1].

Salicylic acid and the subsequent activation of PR genes are necessary for the establishment of SAR in the distant regions of infections [4]. Arabidopsis mutants deficient in the non-expressor of pathogenesis-related 1 (*NPR1*) protein, a key SAR regulator, showed less PR gene expression, which, in turn, increased the susceptibility to pathogens. Various plant species overexpressing Arabidopsis *NPR1* displayed an enhanced disease resistance to pathogens such as *Rhizoctonia solani*, *Erwinia amylovora* and *Erysiphe necator* [5,6,7]. To date, 17 families of PR proteins have been classified and characterized [8]. PR proteins are functionally diverse proteins that are inducible during a pathogen attack and are regulated by signaling compounds such as abscisic acid (ABA), ethylene (ET), jasmonic acid (JA) and salicylic acid (SA) [9,10].

*PR-1a*, the first member of the *PR-1* family, was identified in *Nicotiana tabacum* plants infected with the tobacco mosaic virus. *PR-1* proteins belong to the group of the most abundantly produced proteins during plant defense responses and they are ubiquitous across plant species. *PR-1* proteins are involved in the cell wall thickening and thereby prevent the spread of the pathogens in the apoplast [11]. Apart from the biotic stresses, the role of *PR-1* in abiotic stresses [12] was also reported. During infections, the overexpression of *PR-1* proteins in the apoplast make them potential candidates for antimicrobial activity [13]. An enhanced tolerance to fungi [14], oomycetes [15] and bacterial infections [16] were demonstrated with an overexpression of *PR-1* in the transgenic plants. Apart from their role in various biotic and abiotic stresses *PR-1* and *PR-1*-like proteins have been reported to be involved in plant development, flowering and seed growth [17,18]. *PR-1* proteins are widely reported across the plant kingdom. These are the members of the cysteine-rich secretory protein, antigen 5, and the pathogenesis-related 1 (CAP) protein superfamily. Stress signaling peptides such as CAPE-1 (CAP-derived peptide 1) are embedded within *PR-1* proteins. A CAPE-1 peptide that comprised of the last 11 amino acids from the C-terminus of the *PR-1* protein was reported from tomato plants subjected to wounding and methyl jasmonate treatment [19]. A caveolin-binding motif (CBM) in the CAP region, which is involved in sterol binding, is responsible for antimicrobial activity against oomycetes including *Phytophthora* and *Pythium* species, which require exogenous sterols for basic metabolism. Plants with an enhanced *PR-1* expression are particularly well protected against oomycete pathogens [20]. The in-depth structural and biochemical analysis of *PR-1* proteins can provide greater insights into their function during defense signaling in crop plants.

In the world of spices, black pepper (*Piper nigrum* L., family Piperaceae) is considered to be the king of spices due to its pungent constituent. It is a major additive in many ayurvedic medicinal preparations besides its use as a preservative, a pesticide and a spice. The major hindrance in the *P. nigrum* production is the destructive footrot or quick wilt disease caused by an oomycete, *Phytophthora capsici* [21]. In this work, to extend our knowledge on the defense mechanisms underlying the PR function in *P. nigrum*, we carried out a comprehensive genome-wide analysis and validation of *PR-1* genes from *P. nigrum*. A transcriptome-assisted analysis and expression profiling revealed the differential expression of *PR-1* genes during *P. capsici* infection in *P. nigrum* variety Panniyur-1.

## 2. Materials and Methods

### 2.1. Identification and Analysis of PR-1 Genes from the P. nigrum Genome

PR-1 genes of *Arabidopsis thaliana* were downloaded from the TAIR (The Arabidopsis Information Resource) database (https://www.arabidopsis.org/index.jsp, accessed on 7 January 2021) and a tblastn search against the *P. nigrum* genome assemblies was performed [22]. The coding sequences were translated and aligned by a multiple sequence alignment using the BioEdit Sequence Alignment Editor [23]. The nucleotide and protein sequence conservations of all of the *PnPR-1* candidates were checked using Mega 7. The domain structure prediction was carried out using an NCBI-Conserved Domain Database (CDD) [24].

The molecular weight and pI of *PnPR-1* proteins were estimated by the ExPASy ProtParam tool (https://web.expasy.org/protparam/, accessed on 15 January 2021). The potential signal peptide regions and the cleavage sites were also predicted using a SignalP 5.0 server (http://www.cbs.dtu.dk/cgi-bin, accessed on 15 January 2021). Subsequently, the conserved motifs of *PnPR-1* proteins were predicted using MEME (Multiple Em for Motif Elicitation) (http://meme-suite.org/tools/meme, accessed on 20 January 2021) [25].

### 2.2. GO and KEGG Analysis

Gene ontology (GO) was classified into biological processes, cellular components and the molecular function. The *PnPR-1* genes were analyzed for their role in GO using the PANNZER2 web server (http://ekhidna2.biocenter.helsinki.fi/sanspanz/, accessed on 25 January 2021) [26]. A KEGG (Kyoto Encyclopedia of Genes and Genomes) tool, BlastKOALA (KEGG Orthology and Links Annotation), a web server (https://www.kegg.jp/blastkoala/, accessed on 25 January 2021) [27], was used for the individual characterization of the gene functions.

### 2.3. Secondary and Tertiary Structure Prediction

The secondary structure prediction of the *PnPR-1* proteins was predicted using the Self-Optimized Prediction Method with Alignment (SOPMA) server (https://npsa-prabi.ibcp.fr/cgi-bin/npsa_automat.pl?page=/NPSA/npsa_sopma.html, accessed on 27 January 2021). The predicted 3D structures were built using a Protein Homology/analogY Recognition Engine v2 (Phyre2) server (http://www.sbg.bio.ic.ac.uk/~phyre2/html/page.cgi?id=index, accessed on 27 January 2021) [28]. The CASTp (Computed Atlas of Surface Topography of proteins) tool (http://sts.bioe.uic.edu/castp/calculation.html, accessed on 27 January 2021) was used to predict the active site pockets and topology of the *PnPR-1* protein structures [29].

### 2.4. Role of PnPR-1 in P. capsici-Infected P. nigrum

The control *P. nigrum* (uninfected) leaf transcriptome data (SRA050094) and the *P. capsici*-infected *P. nigrum* leaf transcriptome data (SRX853366) were reanalyzed and the final assembled data were used for the expression studies. The *PnPR-1* sequences curated from the *P. nigrum* genome [22] were mapped to the transcriptome assembly files. The differential regulation of the obtained transcripts was checked using FPKM (fragments per kilobase of transcript per million mapped reads) values.

### 2.5. Plant-Pathogen Infections, Staining and RT-qPCR

Virulent, pure cultures of *P. capsici* and root cuttings of the *P. nigrum* cultivar Panniyur-1 were obtained from the College of Agriculture, Vellayani. *P. capsici* was subcultured every 15 days on potato dextrose agar (PDA) and stored at 28 °C. A 48 h old *P. capsici* culture in PDA was used in this study. *P. capsici* mycelial discs were used to infect the fully-expanded second leaf of the *P. nigrum* plant. The mock treatments were replaced with plain PDA discs. Mock-infected plants were used as a control. The infected leaf samples were collected at 6 h, 12 h and 24 h after infection. The mock samples were collected at 24 h. To detect and visualize the tissue damage in the leaf region after 24 h of pathogen infection, trypan blue staining was performed [30]. Three biological replicates were used for all of the studies.

At each timepoint, the leaf samples were collected and flash frozen in liquid nitrogen and stored at −80 °C until use. Total RNA was isolated from the collected leaf samples using a mirVana miRNA isolation kit (Invitrogen, Cat No: AM1560) according to the manufacturer’s instructions. The quality and the concentration of the RNA samples were checked by using a Colibri Microvolume Spectrometer. The RNA was reverse-transcribed into cDNA using a high-capacity cDNA synthesis kit (Applied Biosystems, Cat No: 4374966). RT-qPCR was carried out using the Applied Biosystems 7900 HT sequence detection system (ABI) using SYBR Green qPCR Master Mix (ABI). Each qPCR reaction was conducted in a 10 μL volume containing 1 μL of diluted cDNA (10 ng/μL), 5 μL of SYBR green and 5 pmol of forward (TCTTGTTGTTCGCAGCCCTAG) and reverse primer (GCGTAATTCCGTGCGTAGGT) with the following conditions: 40 cycles of 95 °C for 15 s DNA denaturation, annealing at 60 °C for 15 s and elongation at 72 °C for 30 s. The 5.8S RNA was used as an endogenous control [31]. The relative quantification was analyzed by the comparative CT method using the formula 2^−∆∆CT^ and the standard deviation was represented as the error bar [32].

### 2.6. Prediction of cis-Acting Regulatory Elements in Promoter Regions

The genomic sequences of the 2.0 kbp upstream of the coding sequence (CDS) region of each *PnPR-1* gene were extracted from the *P. nigrum* genome assembly. The promoter regions of each *PR1* gene were scanned for the presence of functional motifs using Softberry (http://www.softberry.com/berry.phtml?topic=case_study_plants&no_menu=on, accessed on 30 January 2021) and New PLACE (https://www.dna.affrc.go.jp/PLACE/?action=newplace, accessed on 30 January 2021) [33].

## 3. Results

### 3.1. Genome-Wide Identification and Analysis of P. nigrum PR-1 Genes

Eleven potential *PnPR-1* gene candidates were obtained from the genome-wide analysis in comparison with the *A. thaliana* PR genes. The number of exons in each *PnPR-1* gene is one except in Pn2.357, which has two exons. The basic information of the *PnPR-1* genes including the protein sequence length, isoelectric points and molecular weight are listed in Table 1. The length of the *PnPR-1* proteins ranged from 127 to 357 amino acid residues with a molecular weight ranging from 14.38 to 38.49 kDa. The theoretical isoelectric point (pI) data categorized the majority of the *PnPR-1* proteins into basic except Pn21.1032 and Pn36.35 that were acidic. The extreme acidic or basic properties can contribute to distinct functions of each *PnPR-1* gene. Five gene loci for *PnPR-1* were distributed in the *P. nigrum* genome scaffold 23. Furthermore, the signal peptide regions and cleavage sites were also identified in all of the *PnPR-1* protein sequences except Pn11.1637 and Pn31.171 (Table 2). All of the *PnPR-1* genes that were mapped to scaffold 23 had the same signal peptide cleavage positions in between the 24th and 25th amino acids.

**Figure 1:**
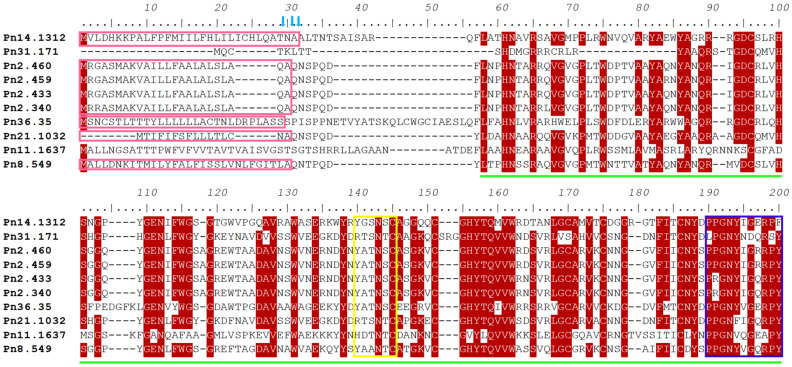
Multiple sequence alignments of the *PnPR-1* protein sequences. The pink colored rectangle boxes indicate the signal peptide regions; the cleavage sites predicted are indicated by the blue arrowheads. The green line indicates the CAP domain structure of ~150 bp. The yellow and blue color rectangles show the caveolin-binding motif (CBM) and CAP-derived peptide (CAPE), respectively.

The multiple sequence alignment of all of the eleven *PnPR-1* proteins revealed highly conserved CAP domain sequences, which further confirmed that these candidates belonged to the CAP superfamily. The CAP domain structure comprises of about 150 amino acids and also include a caveolin-binding motif (CBM) and a CAP-derived peptide (CAPE) motif (Figure 1). A comparison of the *PnPR-1* sequences with various other monocot and dicot plants showed a distinct CAP domain with a CBM and a CAPE (Supplementary Figure S1) in *P. nigrum*. A SignalP analysis revealed about 24 amino acid long signal peptide regions (pink colored boxes) of *PnPR-1* at the N terminal side. The predicted cleavage site is indicated by the arrowhead.

### 3.2. Sequence Conservation of PnPR-1 Genes

**Figure 2:**
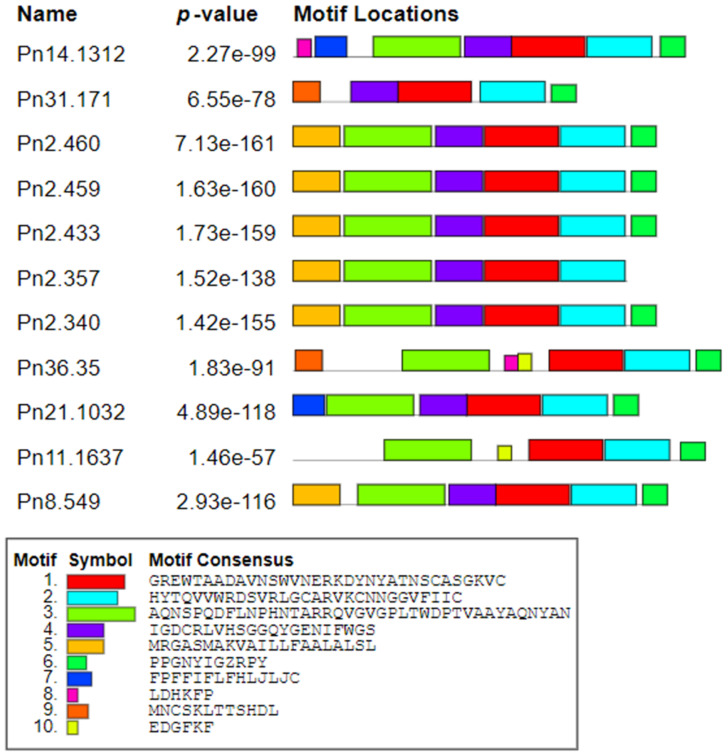
Conserved motifs identified from *Piper nigrum PR-1* protein homologs.

The subsequent phylogenetic analysis of all eleven *PR*-1 nucleotide and protein sequences through the maximum-likelihood method with 1000 bootstraps (Supplementary Figure S2A,B) revealed two main clusters leaving one outnumbered group (Pn21.1032). A total of ten conserved motifs were identified using the MEME server. Motifs 1 and 2 were conserved in all of the deduced *PR-1* proteins whereas motif 3 and motif 6 were conserved in all *PnPR-1* proteins except in Pn31.171 and Pn2.357, respectively. Motif 8, motif 9 and motif 10 were preserved in Pn36.35 along with Pn14.1312, Pn31.171 and Pn11.637, respectively (Figure 2).

A total of 10 conserved motifs were identified. Each color represents different motifs with consensus sequences.

### 3.3. GO and KEGG Pathway Analysis of PnPR-1 Genes

A gene ontology (GO) analysis yielded five biological processes, four molecular functions and four cellular components. Based on the GO enrichment analysis, most of the *PR-1* genes had their role in defense responses and a response to a biotic stimulus in a biological function. In terms of the molecular function, it had protein kinase activity and adenyl nucleotide and purine ribonucleoside-binding activities. The cellular component showed its role in the extracellular region (Table 3). A KEGG pathways analysis categorized its role in the environmental information processing signal transduction pathways such as the MAPK signaling pathway (plant 04016), the plant hormone signal transduction (04075) and the plant-pathogen interaction (04626).

### 3.4. Secondary and 3D Structure of the PnPR-1 Protein

**Figure 3:**
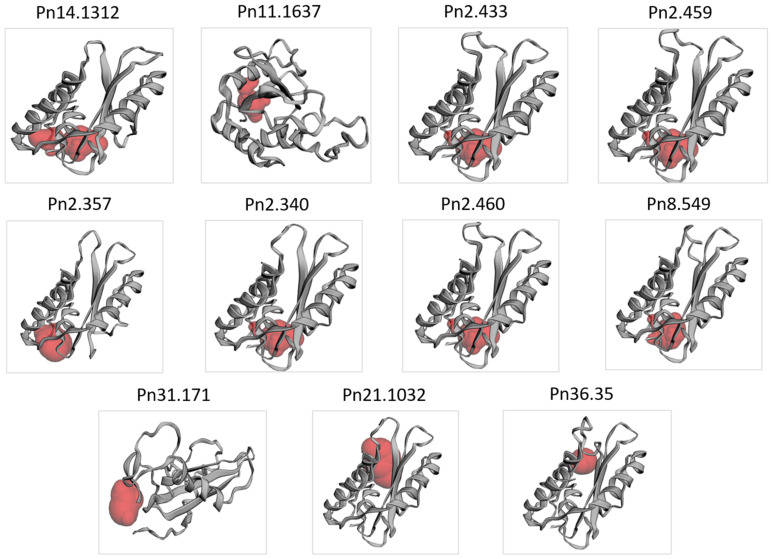
The predicted 3D structures of *PnPR-1* proteins generated using the Phyre2 server and binding pockets identified by the CASTp 3.0 server (red color).

A varied percentage of α-helices (15.6–36.88%), extended strands (10.64–23.78%), β-turns (3.65–5.88%) and random coils (37.42–67.79%) were found in the *Pn-PR1* proteins (Supplementary Figure S3). The relative proportion of the structural features differed among the *PnPR-1* proteins. Pn31.171 and Pn2.357 showed less α-helix structures and more random coils compared with the other counterparts. Despite the 3D structural variations detected among the *PnPR-1* proteins (Figure 3), the binding pockets critical for protein interaction were found in all of the eleven candidates. At the same time, the proportion of disordered regions of *PR-1* proteins ranged from 4.5–28.1%.

### 3.5. Cis-Regulatory Elements of the PnPR-1 Genes

**Figure 4:**
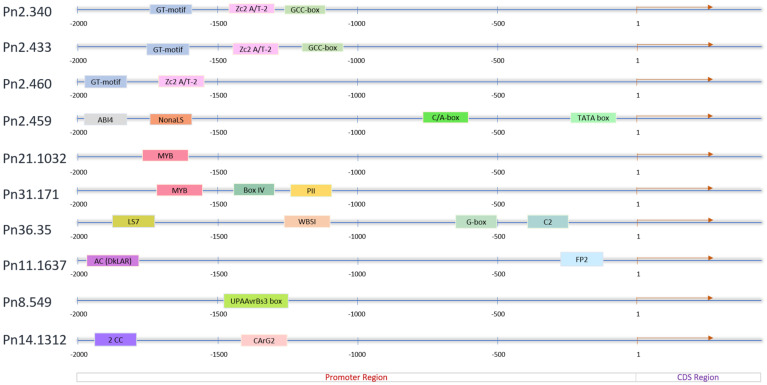
Analysis of the cis-acting elements of the *PnRR-1* promoter regions (2 kb upstream from the CDS region) using Softberry software. Each element is represented with different colors.

The cis-elements were found to be distributed over the 2.0 kb upstream promoter region of the *PnPR-1* genes (Figure 4) except in Pn2.357. The length of these cis-elements varied from 9 to 42 bp in the Softberry database whereas they were 4 to 24 bp in the New PLACE database. This was compared with the length of the cis-elements in *A*. *thaliana* (4–13 bp) and in *O. sativa* (4–10 bp) carried out by using the PlantCare program [34]. Among the ten *PR-1* gene loci, the typical TGA binding site LS7, WBSI, G-box and C-motif were found in the promoter regions of Pn36.35. Meanwhile, the GT motif and Zc2A/T-2 were found in Pn2.340, Pn2.433 and Pn2.460. The hormone signaling elements such as ABI4 and GCC-box were present in Pn2.459, Pn2.340 and Pn2.433, respectively. The stress-responsive MYB was also detected in the promoter regions of the *PnPR-1* genes such as Pn21.1032, Pn31.171 and Pn36.35. Among the 154 cis-elements detected from the New PLACE database, the CAAT box (CAAT), E-box (CANNTG) and DOFCOREZM (AAAG) regions were found to be widely distributed across the *PnPR-1* promoter regions (Supplementary Figure S4).

### 3.6. Expression of PR-1 Genes during P. capsici Infection in P. nigrum

**Figure 5:**
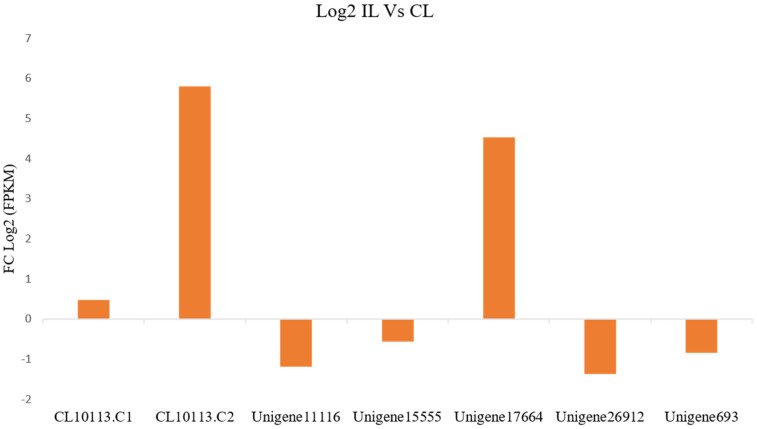
Expression analysis of the *PnPR-1* transcripts in *Phytophthora capsici*-infected *P. nigrum* using FPKM (fragments per kilobase of transcript per million mapped reads) values. From the left, the *PnPR-1* transcripts are designated as CL10113.C1, CL10113.C2, Unigene11116, Unigene15555, Unigene17664, Unigene26912 and Unigene693. Positive and negative numbers on the *X*-axis indicate the fold changes in the Log_2_ gene expression levels.

The assembled transcriptome of the RNA-seq data from the control (NCBI-SRA050094) and the *P. capsici*-infected *P. nigrum* (NCBI-SRX853366) plants revealed 60,437 transcripts. From the assembled data, seven transcripts of the *PnPR-1* genes were mapped to the *P. capsica*-*P. nigrum* interaction pathway. The transcript lengths ranged from 391–1015 bps. A differential expression of these transcripts between the control leaf (CL) and the infected leaf (IL) was assessed from their corresponding FPKM values. CL10113.C1/2 and Unigene17664 were mapped to the Pn23 and Pn8 scaffolds, respectively, and were significantly upregulated (*p*-value < 0.01) in the IL compared with the CL. Meanwhile, Unigene11116, Unigene15555, Unigene26912 and Unigene693 were significantly downregulated (*p*-value < 0.01) in the IL compared with the CL (Figure 5).

### 3.7. Trypan Blue Staining and Microscopic Detection of P. capsici Infection

**Figure 6:**
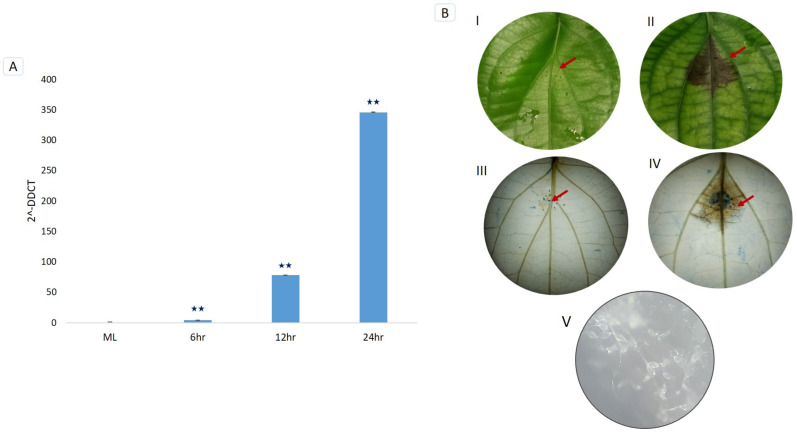
(**A**) Temporal expression validation of CL10113.C2 *PnPR-1* gene in *Phytophthora capsici*-infected *Piper nigrum* plants by RT-qPCR. ML (mock-infected leaf) 6 hpi (hours post-infection), 12 hpi and 24 hpi. Asterisks indicate significant changes (*p*-value < 0.001). (**B**) Mock and *Phytophthora capsici*-infected *Piper nigrum* leaves after 24 h of infection. (I) Mock-infected leaf. (II) *Phytophthora capsici*-infected *Piper nigrum* leaf. (III) Trypan blue-stained mock-infected leaf. (IV) Trypan blue-stained infected leaf. (V) *Phytophthora capsici* spores developed from the infected tissues. Red colored arrows indicate the infected regions.

The development of necrotic lesions was detected on *P. nigrum* leaves after the *P. capsici* infection. As the lesion size progressed with the infection, the pathogen spores were profusely developed from the infected tissues. *P. capsici*-induced cell death on *P. nigrum* leaves was detected using trypan blue staining. The infected tissue was stained in blue whereas the viable cells were colorless (Figure 6B).

### 3.8. RT-qPCR Validation of PnPR-1 Genes

We validated the relative expression of the remarkably upregulated *PnPR-1* gene (CL10113.C2; Figure 5) using RT-qPCR from *P. capsici*-infected *P. nigrum* and mock control (ML) plants. The temporal expression at 6 h, 12 h and 24 h of *P. capsici* infections revealed a differential expression of the *PnPR-1* (CL10113.C2) gene. A significant (>0.001) upregulation of the *PnPR-1* gene (CL10113.C2) was observed in the leaves at 6 hpi, 12 hpi and 24 hpi compared with the mock control plants (Figure 6A).

## 4. Discussion

PR proteins are defense-related signaling molecules induced by phytopathogens that play a vital role in resisting the entry of the invading pathogen. PR proteins have been classified into many families based on their function, molecular weight, amino acid sequence and other properties [35]. In tobacco, PR proteins were initially classified as five major classes (PR1, PR2, PR3, PR4 and PR5) [36]. However, later studies in tobacco and the tomato have grouped them into 11 families [37]. The members belonging to the PR family can be either acidic or basic. Basic PR proteins are located intracellularly in the vacuole regions and are constitutively expressed to some extent and are also induced by stress signals whereas acidic types are produced extracellularly and only triggered by specific stress signals [38]. In our current study, both acidic and basic *PR-1* proteins, which have a critical role during *P. nigrum*-*Phytophthora* interactions, were identified. The majority detected were basic in nature; this was the same as in the case of the *PR1* proteins studied in *S. lycopersicum* during drought stress [12]. Contrasting to this, a greater number of acidic *PR1* genes were also reported from rice, where among 12 *PR1* rice protein candidates seven were acidic in nature [39]. In *Glycine max* during multiple biotic and abiotic stresses among 24 *PR1* proteins, 15 were detected as acidic [40].

A KEGG orthology analysis revealed *P. nigrum PR-1* genes mapped to the plant-pathogen interaction (04626), MAPK signaling pathway (04016) and plant hormone signal transduction (04075). The *PR-1* family mainly possesses antifungal and anti-oomycete activities [35]. The overexpression of PR1 or similar proteins in various plants leads to an enhanced disease resistance to a wide variety of pathogens [39], especially the oomycetes [15]. The anti-oomycete properties of PR-1 proteins such as P14c and PR-1 were demonstrated against the sterol auxotroph pathogen *Phytophthora brassicae* [20].

As previously reported [13,20], *P. nigrum PR-1* proteins possess the CAP tetrad, the CBM involved in sterol binding [41] and the CAPE involved in plant immune signaling [19]. The ability of the *PR-1* family of proteins to bind sterols contributes towards their antimicrobial activity towards the *Phytophthora* species, a major plant pathogen belonging to the sterol auxotroph [20]. The CAPE-1 peptide has the consensus motif PxGNxxxxxPY, which is conserved between the monocots and dicots. A highly conserved and distinct similarity in the domains of the protein structure was observed in *PnPR-1* proteins, which might account for a general strategy in responding to various biotic stresses as reported in other studies [42]. The role of the CAPE-1 peptide in the defense signaling was demonstrated in a previous study on the *Pseudomonas syringae* pv tomato (Pst) strain DC3000 interaction in tomatoes. A diverse set of defense-related genes was induced in the tomato plants pretreated with the CAPE-1 peptide. Furthermore, a non-canonical pathway other than PAMP-triggered immunity (PTI) signaling was suggested for the CAPE-1 mediated defense responses as the CAPE-1 did not induce the WRKY transcription factor 53 (WRKY53) [19]. The pathogen effector ToxA and Tox3 proteins from *Parastagonospora nodorum* were found to interact with wheat *PR-1* proteins [43,44]. Promoters are the regulators of a gene at the transcriptional level [45]. Various computational methods are thoroughly being used for the identification of different cis-elements in the promoter region that are responsible for the regulation of genes [46]. A range of cis-elements was predicted from *P. nigrum PR-1* genes, which were likely related to the regulation of the plant growth, development and response to various stresses. A high frequency of the CAAT box (CAAT), E-box (CANNTG) and DOFCOREZM (AAAG) regions was found in the *PnPR-1* promoter regions. A high occurrence of the CAAT box was previously reported in *A. thaliana* PR proteins [34]. As previously reported, hormone-regulating sequence motifs [45] such as LS7, GCC-box, ABA-responsive elements (ABREs; also termed as G-box) [47] and ABI4 were detected from the upstream of certain *PnPR-1* genes. LS7, which contains a TGA binding site, has been reported to be the key activator of *PR-1* expression, NPR1 [48]. The ethylene-responsive factor binds to the GCC-box (ethylene-responsive element) and responds to various biotic and abiotic factors in Arabidopsis [49]. The ABA-insensitive-4 (ABI4) transcription factor was reported to be involved in ascorbate-dependent plant growth [50]. AC elements in the promoter region of the Leucoanthocyanidin reductase gene in the Proanthocyanidin pathway promoter harbors the binding site for MYB2 Myb-like transcription factors [51]. Subsequently, the WER-binding site (WBSI) and NonaLS are also reported in the *PnPR-1* promoter region. WBSI was detected in the *CAPRICE* (*CPC*) promoter of Arabidopsis. WEREWOLF (WER) is a MYB protein and gene transcription activator during the specification of epidermal cell fates [52]. NonaLS (Nona-like sequence, GATCGGACG) is the positive cis-acting element of histone *H1* genes in wheat, tomato and Arabidopsis [53]. The induction of both biotic- and abiotic-responsive cis-acting regulatory elements in *PnPR-1* indicates that these genes play a key role in regulating resistance against *P. capsici* and other abiotic stresses in *P. nigrum*.

Even though *PR-1* proteins belong to the group of abundant proteins expressed in the plant-pathogen interaction, all of the members of the family were not uniformly upregulated [13]. Likewise, a transcriptome-assisted analysis revealed a high upregulation of two basic *PR-1* genes (pI > 7.35) such as CL10113.C1/2 and Unigene17664 in *P. nigrum* upon *P. capsici* infection. As *PR-1* genes belong to the group of multigene families, they differ widely in their properties. *PR-1* expression levels rise both transcriptionally and translationally upon pathogen infections [54,55]. Consistent with previous studies, the upregulation of *PnPR-1* transcripts such as CL10113.C1, CL10113.C2 and Unigene17664 were detected during *P. capsici* infection in *P. nigrum*. The qRT-PCR expression studies also validated the drastically increased CL10113.C2 expression pattern at 24 hpi (Figure 6A). The significant upregulation of *PR1* in *P. nigrum*-infected *P. capsici* showed similar expression patterns in the case of *Brassica juncea* and *Erysiphecruciferarum* pathogen interaction where the expression of *PR1* was strongly upregulated [56]. This resembles the mechanism of PR genes being upregulated following pathogen infection, indicating that *P. capsici* actively works in manipulating the *P. nigrum* host defenses. In addition to the host defense response during pathogen infection, *PR-1* proteins were also reported to have a role in abiotic stress stimuli [57,58,59].

The trypan blue staining clearly showed the necrotic region as the defense response of the host plant to the pathogen and its further effect to inhibit the growth of the pathogen to the surrounding regions (Figure 6B). This was, in turn, proved by the significant upregulation of the *PnPR-1* genes at 24 h post-infection in the leaf samples using qPCR experiments. To date, only a few studies have been carried out on the role of *PR* proteins in *P. nigrum* or related Piperaceae species. The activity of PR protein chitinase, β-1,3-glucanase and their related enzymes were reported in *P. capsici*-infected *P. nigrum* plants [60,61,62]. The present study contributes a significant advancement in the understanding of the molecular function of *PR-1* proteins in *P. nigrum*. *PnPR-1* genes are found to have a key role in the early defense such as PTI towards *P. capsici* infection in Panniyur-1 plants. It may be possible that the key genes in the subsequent effector-triggered immunity act as critical players of the defense response in Panniyur-1 plants. Therefore, future studies on the identification of the potential *P. capsici* effectors coupled with *PR-1* functional studies will ascertain the in-depth mechanisms of the defense signal amplification and anti-oomycete properties of these enigmatic proteins in *P. nigrum*.

## 5. Conclusions

The genome-wide survey identified eleven *P. nigrum PR-1* gene homologs mapped to seven distinct genome scaffolds. A subsequent transcriptome analysis of *P. capsici*-infected *P. nigrum* plants showed the expression of *PR-1* genes from all of the mapped loci. Our study revealed the differential regulation of *PR-1* gene candidates in *P. capsici*-infected *P. nigrum* plants. A significant upregulation was detected for the transcripts of certain *PnPR-1* genes such as CL10113.C2 and Unigene17664. A detailed in silico analysis revealed cis-regulatory elements such as phytohormone-responsive transcription activators in the promoter regions. The structural analysis revealed similar binding pockets in the predicted 3D structures of all *PnPR-1* proteins except Pn31.171. The differential expression of certain *PnPR-1* homologs revealed their crucial role during the early defense response in the *P. capsici-P. nigrum* interaction. Further in-depth functional studies on *PnPR-1* genes, promoter cis-regulatory elements and the pathogen-specific effectors can provide the exact molecular mechanism of the susceptibility/tolerance of *P. nigrum* cultivars to Phytophthora infection, which, in turn, can contribute towards the novel disease protection strategies in *P. nigrum* plants.

## Figures and Tables

**Table 1:** Sequence characteristics and physio-chemical properties of the *PR-1* proteins in *Piper nigrum*.

Genome CDS Id	Genome Scaffold No.	Exon No.	Start	Stop	Strand	Protein Length (AA)	Molecular Weight (kDa)	Theoretical pI	
Pn2.460	Pn23	1	1315655	1316146	−	155	17.09488	4.82	Acidic
Pn2.459	Pn23	1	1320928	1321419	−	192	21.91361	5.8	
Pn2.433	Pn23	1	1493563	1494054	−	168	18.35074	7.58	Basic
Pn2.357	Pn23	2	2226595	2229193	−	127	14.37881	7.62	
Pn2.340	Pn23	1	2402850	2403341	−	185	19.80756	9.1	
Pn21.1032	Pn3	1	3202437	3202904	+	163	17.85002	9.15	
Pn31.171	Pn15	1	26854949	26855332	−	163	17.87807	9.3	
Pn36.35	Pn25	1	5754975	5755553	−	163	17.88005	9.3	
Pn11.1637	Pn4	1	33322945	33323502	−	176	19.63337	9.37	
Pn8.549	Pn8	1	25779763	25780269	−	163	17.96421	9.44	
Pn14.1312	Pn14	1	5932334	5932864	+	357	38.49596	11.3	

**Table 2:** Signal peptide region detected from the *PnPR-1* proteins.

Genome Id	Cleavage Site Position	Sequence Position	Probability	Protein Type	Signal Peptide (Sec/SPI)	Other
Pn21.1032	16 and 17	CNA-QN	0.9541	Likelihood	0.9981	0.0019
Pn2.340	24 and 25	AQA-QN	0.8849	Likelihood	0.9984	0.0016
Pn2.357	24 and 25	AQA-QN	0.8898	Likelihood	0.9986	0.0014
Pn2.433	24 and 25	AQA-QN	0.8892	Likelihood	0.9987	0.0013
Pn2.459	24 and 25	AQA-QN	0.8898	Likelihood	0.9986	0.0014
Pn2.460	24 and 25	AQA-QN	0.8892	Likelihood	0.9987	0.0013
Pn36.35	29 and 30	ASS-SP	0.6292	Likelihood	0.9813	0.0187
Pn14.1312	31 and 32	TNA-AL	0.4399	Likelihood	0.7324	0.2676
Pn8.549	30 and 31	TLA-QN	0.3454	Likelihood	0.5106	0.4894
Pn31.171	-	-	-	Likelihood	0.0029	0.9971
Pn11.1637	-	-	-	Likelihood	0.2691	0.7309

**Table 3:** The gene ontology (GO) term distribution of *PnPR-1* proteins.

GO ID	GO Domain	Function Description
GO:0006952	Biological process	Defense response
GO:0009607	Biological process	Response to biotic stimulus
GO:0048544	Biological process	Recognition of pollen
GO:0006468	Biological process	Protein phosphorylation
GO:0010274	Biological process	Hydrotropism
GO:0004672	Molecular function	Protein kinase activity
GO:0030554	Molecular function	Adenyl nucleotide binding
GO:0035639	Molecular function	Purine ribonucleoside triphosphate binding
GO:0032555	Molecular function	Purine ribonucleotide binding
GO:0005576	Cellular component	Extracellular region
GO:0016020	Cellular component	Membrane
GO:0031224	Cellular component	Intrinsic component of membrane

## Data Availability

Control *P. nigrum* (uninfected) leaf transcriptome data and *P. capsici*-infected *P. nigrum* leaf transcriptome data can be accessed from NCBI SRA, accession numbers SRA050094 and SRX853366, respectively.

## Supplementary Materials

The following are available online at https://www.mdpi.com/article/10.3390/genes12071007/s1, Figure S1: The amino acid sequences from *Piper nigrum*, *Nymphaea colorata*, *Elaeis guineensis*, *Cinnamomum micranthum*, *Gossypium tomentosum*, *Musa* ABB, *Amborella trichopoda* and *Phoenix dactylifera* were aligned using Geneious bioinformatics software (https://www.geneious.com/, accessed on 28 January 2021) with default settings. The conserved domains are highlighted in different colors. The CAP domain structure with a caveolin-binding motif (CBM) and a CAP-derived peptide (CAPE) are shown in red and pink boxes; Figure S2: Phylogenetic analyses of *Piper nigrum PR-1* nucleotide and protein sequences. The phylogenetic tree was constructed using MEGA 7.0. by the maximum-likelihood (ML) method with 1000 bootstrap replicates and default parameters. The *PnPR-1* family genes were divided into two major groups, groups I and II; Figure S3: Secondary structure analyses of *PnPR-1* proteins; Figure S4: Analysis of cis-acting elements in *PnPR-1* promoters using the New PLACE online server. The number of elements in each gene is represented in data bars.
