# Supplementary material for: A Genome-Wide Analysis of Pathogenesis-Related Protein-1 (PR-1) Genes from Piper nigrum Reveals Its Critical Role during Phytophthora capsici Infection
Source: Genes (Basel). 2021 Jun 30;12(7):1007. doi: 10.3390/genes12071007 (PMC8303604; doi:10.3390/genes12071007)
Supplement: Supplementary file 1 [file genes-12-01007-s001.zip › genes-1225917-supplementary.pdf]

A Genome-Wide Analysis of Pathogenesis-Related Protein-1 (PR-1) Genes from *Piper nigrum* Reveals Its Critical Role During *Phytophthora capsici* Infection

Divya Kattupalli †, Srinivasan Asha † and Eppurathu Vasudevan Soniya *

Supplementary Figures

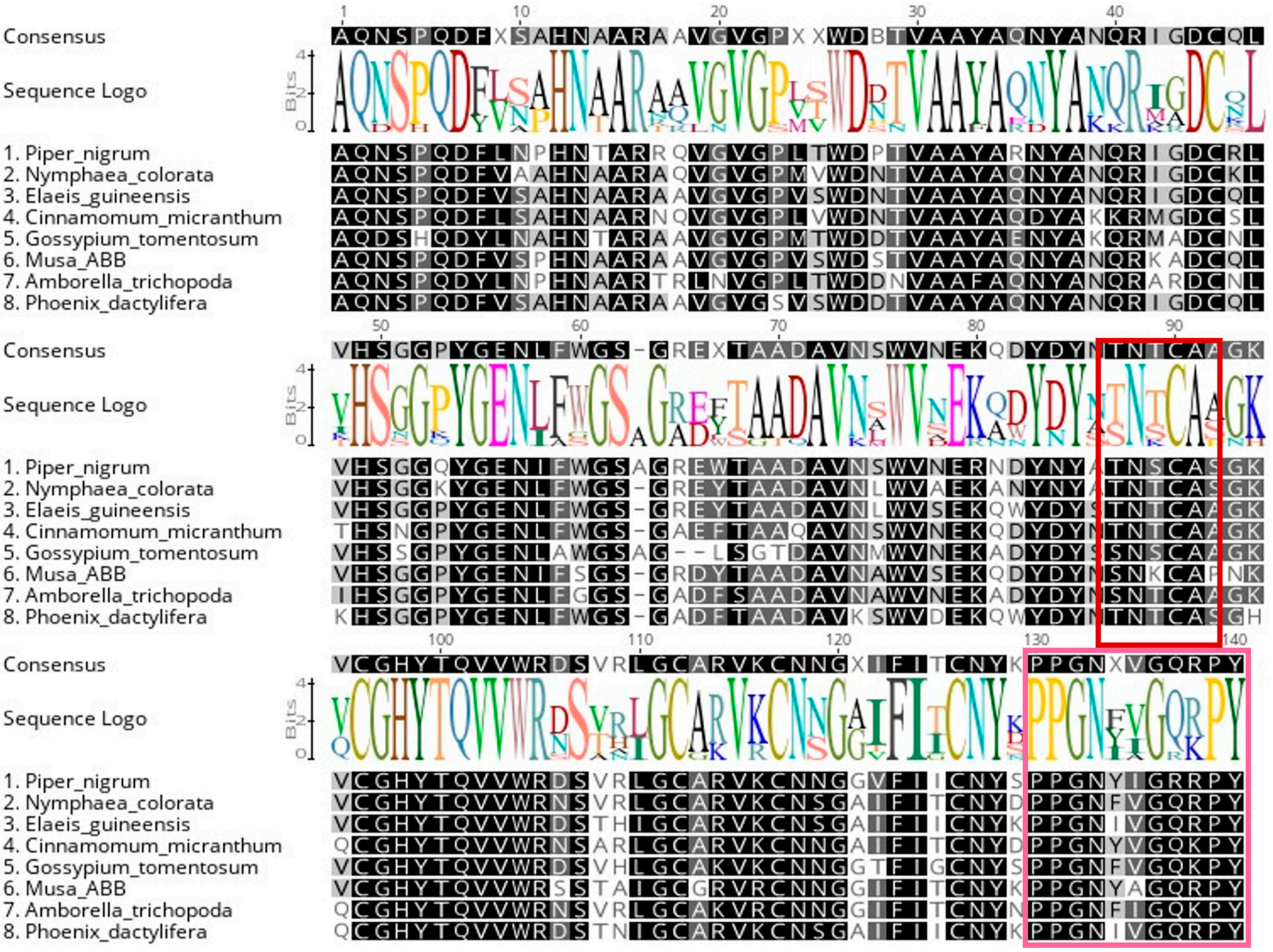

Supplementary Figure S1. The amino acid sequences from *Piper nigrum*, *Nymphaea colorata*, *Elaeis guineensis*, *Cinnamomum micranthum*, *Gossypium tomentosum*, *Musa* ABB, *Amborella trichopoda* and *Phoenix dactylifera* were aligned using Geneious bioinformatics software (https://www.geneious.com/ Accesssed on January 28th 2021) with default settings. The conserved domains are highlighted in different colors. The CAP domain

structure with a caveolin-binding motif (CBM) and a CAP-derived peptide (CAPE) are shown in red and pink boxes.

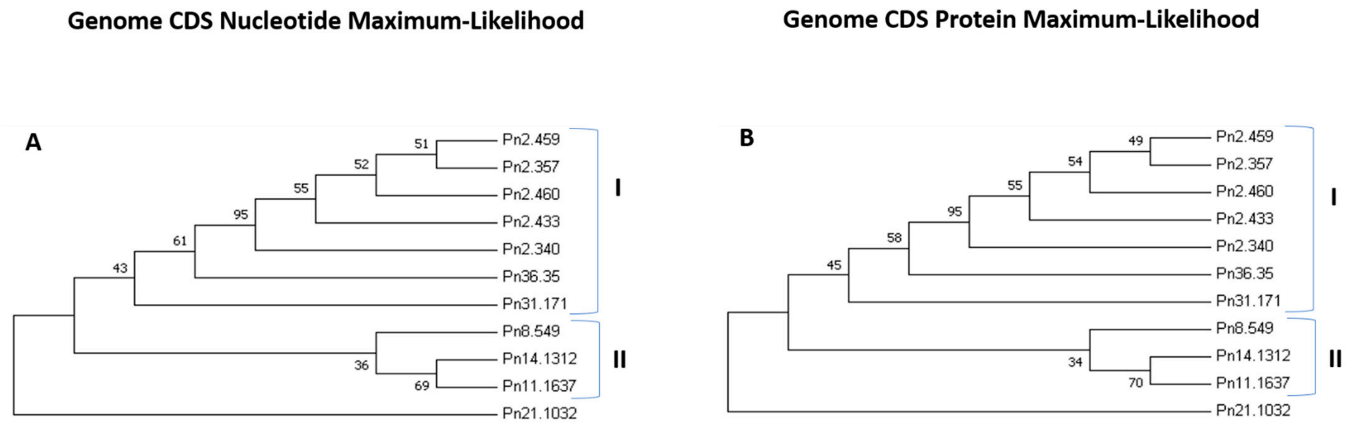

Supplementary Figure S2. (**A**, **B**) Phylogenetic analyses of *Piper nigrum PR-1* nucleotide and protein sequences. The phylogenetic tree was constructed using MEGA 7.0. by the maximum-likelihood (ML) method with 1000 bootstrap replicates and default parameters. The *PnPR-1* family genes were divided into two major groups, groups I and II.

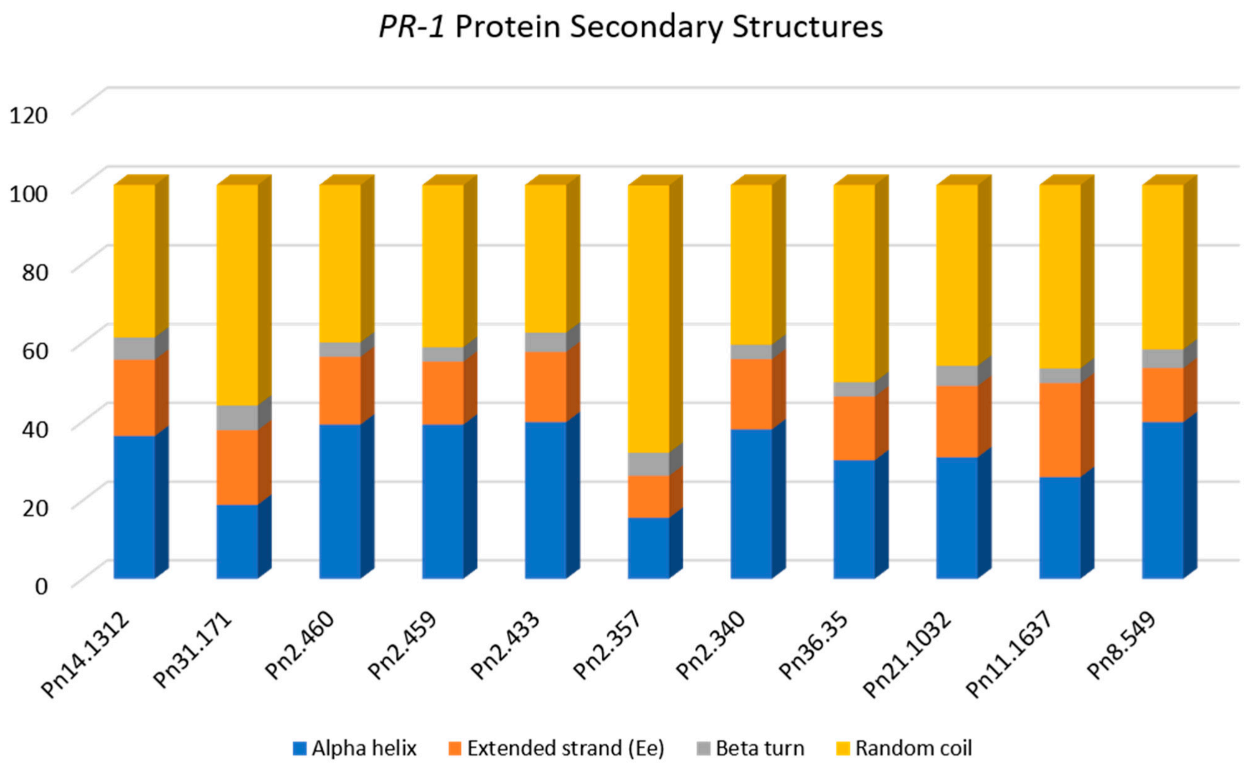

Supplementary Figure S3. Secondary structure analyses of *PnPR-1* proteins.

Factor or Site Name	Sequences	Pn2.460	Pn2.433	Pn2.459	Pn8.549	Pn2.340	Pn2.357	Pn14.1312	Pn36.35	Pn21.1032	Pn11.1637	Pn31.171
DOFCOREZM	AAAG											
NODCON1GM	AAAGAT											
AACACOREOSGLUB1	AACAAAC											
POLASIG1	AATAAA											
POLASIG3	AATAAT											
POLASIG2	AATTAAA											
ACGTATERD1	ACGT											
POLLEN1LELAT52	AGAAA											
BOXIINTPATPB	ATAGAA											
ROOTMOTIFTAPOX1	ATATT											
RAV1AAT	CAACA											
CIACADIANLELHC	CAANNNNATC											
CAATBOX1	CAAT											
EBOXBNNAPA	CANNTG											
CATATGGMSAUR	CATATG											
MYCATERD1	CATGTG											
CCAATBOX1	CCAAT											
MYBCORE	CNGTTR											
NODCON2GM	CTCTT											
CARGCW8GAT	CWWWWWWWWG											
GT1GMSCAM4	GAAAAA											
EECCRCAH1	GANTTNC											
GATABOX	GATA											
IBOXCORE	GATAA											
MYBST1	GGATA											
GT1CORE	GGTTAA											
GT1CONSENSUS	GRWAAW											
CURECORECR	GTAC											
GTGANTG10	GTGA											
ARR1AT	NGATT											
SEF4MOTIFGM7S	RTTTTTR											
CBFHV	RYCGAC											
TAAAGSTKST1	TAAAG											
MYBGAHV	TAACAAA											
GAREAT	TAACAAR											
AMYBOX1	TAACARA											
HDZIP2ATATHB2	TAATMATTA											
TATABOX2	TATAAAT											
-10PEHVPSBD	TATTCT											
WRKY71OS	TGAC											
ASF1MOTIFCAMV	TGACG											
WBOXHVISO1	TGACT											
WBOXNTERF3	TGACY											
BIHD1OS	TGTCA											
TGTCACACMCUCUMISIN	TGTCACA											
TATABOX5	TTATTT											
WBOXATNPR1	TTGAC											
CGCGBOXAT	VCGCGB											
CACTFTPPCA1	YACT											
SEBFCONSSTPR10A	YTGTCWC											

Supplementary Figure S4. Analysis of cis-acting elements in *PnPR-1* promoters using the New PLACE online server. The number of elements in each gene is represented in data bars.
